# *FOXE1* Mutation Screening in a Case with Cleft Lip, Hypothyroidism, and Thyroid Carcinoma: A New Syndrome?

**DOI:** 10.1155/2017/6390545

**Published:** 2017-08-27

**Authors:** Hugo Mendieta-Zerón, Angélica Jiménez-Rosales, Carlos Jhovani Pérez-Amado, Silvia Jiménez-Morales

**Affiliations:** ^1^Ciprés Grupo Médico S.C. (CGM), Felipe Villanueva 700, Col. Morelos, 50120 Toluca, MEX, Mexico; ^2^Universidad Autónoma del Estado de México (UAEMex), Felipe Villanueva Sur 1209, Col. Rancho Dolores, 50170 Toluca, MEX, Mexico; ^3^Programa de Maestría en Ciencias Bioquímicas, Universidad Nacional Autónoma de México (UNAM), Mexico City, Mexico; ^4^Instituto Nacional de Medicina Genómica (INMEGEN), Mexico City, Mexico

## Abstract

A 26-year-old woman is referred to the Internal Medicine consultation due to increases in laboratory studies associated with Papillary Thyroid Carcinoma (PTC) that was confirmed by histopathological studies. Her clinical history revealed that, at 3 months of age, she was successfully treated with surgery for cleft lip (CL) and at the age of 24 years was diagnosed with hypothyroidism. Single nucleotide polymorphisms (SNPs) in* FOXE1* and its promoter regions have been associated with various etiologies related to the thyroid, including orofacial clefting, specially cleft palate (CP) and CL, hypothyroidism (HT), and thyroid cancer. The association of CL, HT, and PTC might be component of a new syndrome; however* FOXE1* coding region, which has been involved with these entities, has not exhibited mutations or SNPs. Further study of other genes may help in better characterization of the possible syndrome.

## 1. Introduction

Transcription factors NKX2-1, FOXE1, HHEX, and PAX8 are involved in cellular differentiation during embryogenesis. They play a critical role in the morphogenesis, differentiation, and maintenance of the thyroid gland. The* FOXE1* gene encodes for a transcription factor protein that is expressed from the embryonic stage in the thyroid primordium until the development of the thyroid gland by the regulation of thyroid promoters [1]. Single nucleotide polymorphisms (SNPs) in this gene and its promoter regions have been associated with various etiologies related to the thyroid, including orofacial clefting, especially cleft palate (CP) and cleft lip (CL), hypothyroidism (HT), and thyroid cancer (TC).

CP and CL are common birth defects, particularly in Asian and Native American population, which have the highest rates of prevalence, in contrast to African population, which has the lowest. Both have a complex etiology, in that they possess multigenetic and multifactorial causes that have not been elucidated. However, research and mapping of the 9q22-q33 guided the associations with several SNPs located in the* FOXE1* locus [2]. Actually, the genotypes involving the commonest alleles of rs3758249 (GG) and rs4460498 (CC) were the most associated with CP in Caucasian and Asian derived populations [3–5]. To note, both SNPs are located in a high linkage disequilibrium (LD) region, which also harbors the rs1867277 (−283G>A FOXE1) SNPs that have been reported as functional in thyroid cancer [6].

HT is the most common thyroid disorder. It is characterized by low production of thyroid hormones T3 and T4. Through Genome-Wide Association Studies (GWAS), various polymorphisms, including SNP located in the* FOXE1* gene, have been identified with problems in the formation and differentiation of the thyroid, producing a predisposition to the disease [5].

TC is the most common endocrine malignancy, with a strong genetic component that has been shown to extend beyond the nuclear family. Histologically, it is classified as Papillary (PTC), Follicular (FTC), and Medullary (MTC) Carcinomas, and undifferentiated anaplastic thyroid carcinomas, and studies have suggested that gender, age, tumor size, histologic type, tumor infiltration, and vascular/lymphatic invasion affect clinical outcome and treatment options [13]. Evidence in multiple ethnicities has associated SNP with the same LD region as* FOXE1* with PTC and MTC [1, 6]. The case described here represents unexpected combination of CL, HT, and PTC.

## 2. Case Report

**Figure 1:**
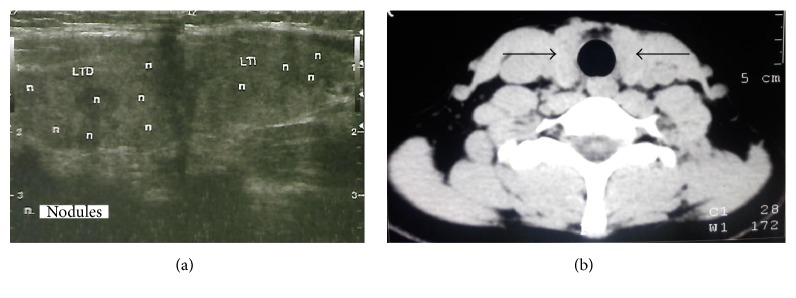
The imaging studies showed multinodularity (US) (a) and thyroid growth (CAT) (b). Arrows in (b) show an enlarged thyroid gland.

**Figure 2:**
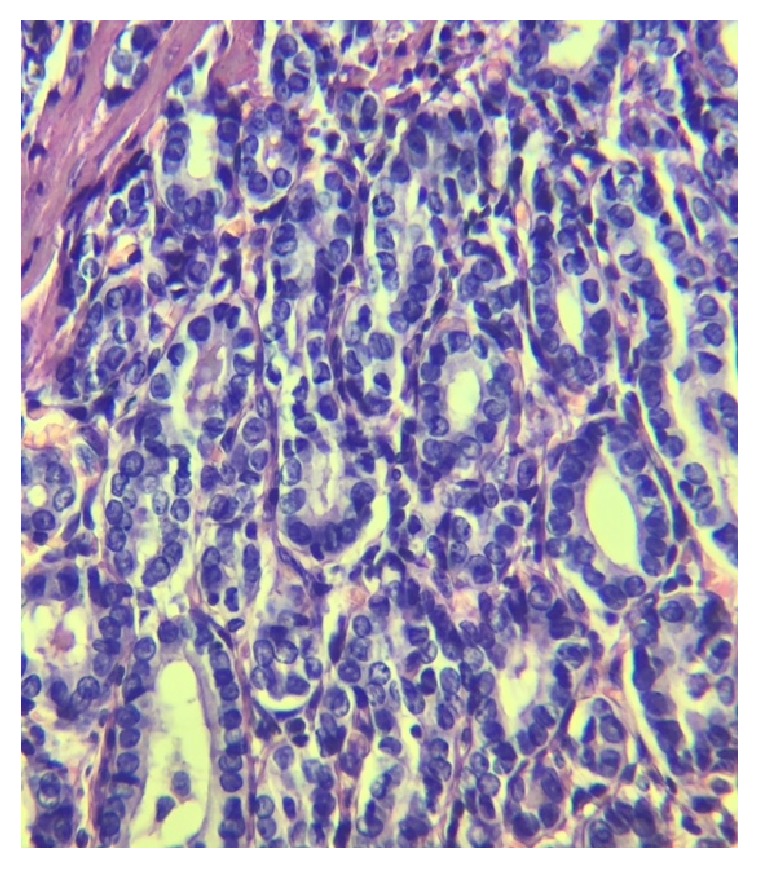
Thyroid fine needle aspiration (FNA) biopsy.

A 26-year-old woman from Jalisco (Mexico) is referred to the Internal Medicine consultation due to increases in laboratory studies associated with PTC. Her clinical history revealed that, at 3 months of age, she was successfully treated with surgery for CL, at 14 years of age underwent a cholecystectomy, and at the age of 24 years was diagnosed with HT. In April 2016, her clinical examinations revealed that she had anti-thyroglobulin antibodies (>4,000 *μ*g/L) and anti-peroxidase antibodies > 42 IU/mL (normal reference value, 34). Imaging studies demonstrated multinodularity (Ultrasound [US]) (Figure 1(a)), discrete thyroid growth (Figure 1(b)) and the thyroid fine needle aspiration (FNA) biopsy corroborated PTC Bethesda Class V (Figure 2).

**Figure 3:**
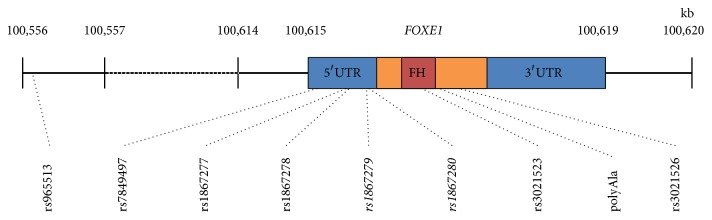
Diagram of the* FOXE1* region marking some single nucleotide polymorphisms (SNPs) reported. The 5′ and 3′ untranslated regions (UTR) are marked in a blue box, the* FOXE1* gene exon is marked in an orange box, the forkhead region is marked in a red box, and the SNPs present in our patient are marked in italic.

Based on the contribution of* FOXE1* mutations and SNPs variants (Figure 3) in the development of the CL, HT, and PC, we sequenced the exon 1 of this gene (coding region and 3′UTR) in order to identify mutations or SNPs potentially associated with the patient's diseases. At the Research Laboratory of Ciprés Grupo Médico S.C. (CGM), Genomic DNA was isolated from peripheral white blood cells using the DNA purification kit (Quick-DNA Miniprep Plus Kit, Zymo Research Corp) according to the manufacturer's instructions. At the INMEGEN Exon 1 of* FOXE1* gene was analysed using the primers described in Table 1. PCR was performed in a final volume of 50 *μ*l, using 50 ng of genomic DNA, 2.5 pmoles of each primer, 2 mM of each dNTP, 1 U of AmpliTaq Gold DNA Polymerase, 4 mM MgCl_2_, and 10x PCR Gold buffer (Applied Biosystems provided polymerase, MgCl_2_, and buffer). Cycling conditions for PCR consisted of a first denaturation step of 95°C for 10 min, followed by 35 cycles of denaturation at 95°C for 20 s, annealing at 60°C for 20 s, and extension at 72°C for 30 s, followed by a final extension at 72°C for 7 min. The runs were carried out on a GeneAmp PCR system 9700 (Applied Biosystems). PCR products were sequenced directly with a DNA Sequencing Kit with Big Dye Terminator on an automated ABI 3730XL sequencer (Applied Biosystems, Foster City, CA, USA) and data analyses were performed by Lasergene software (DNASTAR Inc.). Neither mutations nor SNPs were found in* FOXE1* coding region. Two SNPs (rs1867279 and rs1867280) that have already been associated with CP were found in a homozygote wild type genotype. Additionally, we found the rs71369530 (Ala-14) in homozygote fashion. Functional prediction analysis using the* SNPinfo* Web Server (https://snpinfo.niehs.nih.gov/snpinfo/snpfunc.html) showed that only the ancestral alleles of rs1867279 and rs1867280 might recruit the E2F and NFKB transcription factors, respectively.

## 3. Discussion

### 3.1. Generalities

Several studies in genetics have shown that HT, TC, and isolated CL and CP are related to the* FOXE1* locus.* FOXE1* (Forkhead box E1; UniGene accession number Hs.159234) is an intron-less single exon gene that encodes transcription factor FOXE1 (or TTF-2). FOXE1 is an 373 amino acid protein (38 kDa) that contains a forkhead winged helix DNA binding domain and a polyalanine (polyAla) tract of variable length (11–19 Ala, but 14-Ala is most abundant) and negatively regulates thyroglobulin (TG) and Thyroid Peroxidase (TPO) expression, which regulates, in conjunction with NKX2-1 (Homeobox protein or TTF-1), PAX8, and HHEX, thyroid differentiation and function [9, 17].

RNA in situ hybridization studies in zebrafish in the fish ortholog of* FOXE1*,* foxe1*, indicated that during the postfertilization development, the gene is early expressed in the nervous system, afterward in the oral epithelium and thyroid gland, later in the developing heart, and, in the final stages, in the pharyngeal arch epithelium [5]. Moreover, phenome-wide association studies of genotypes of electronic medical records in DNA biobanks have associated the* FOXE1* locus with thyroiditis, nodular and multinodular goiters, and thyrotoxicosis, among others [18], indicating that mutations in this gene and its promoters exert an important effect on normal thyroid function.

### 3.2. Orofacial Clefts

Orofacial clefts (OC) comprise clefts, disruption, or gaps in structures such as lips, palate, eyes, and nose and arise from failure of normal craniofacial development, in which cells grow, migrate, and differentiate in a coordinated fashion to fuse together the lateral, medial, and maxillary processes. Moreover, given the different developmental origins of lip and palate, these are classified in CL with or without CP [19].

Isolated CL and CP are common genetic defects with a strong genetic component that has not been completely elucidated, and approximately 70–80% of CL/CP that are nonsyndromic (absence of additional structural or cognitive abnormalities) represent complex human disorders with the interaction of genetic risk factors and environmental exposures that increase susceptibility [19]. Evidence suggests that rare DNA variations in several genes and SNPs (many of these are present in noncoding DNA) are linked to CL and CP etiology [3, 8].

### 3.3. Relation of* FOXE1* with Orofacial Clefts

Genome-wide association studies (GWAS) have related* FOXE1* with CL and CP in different population, within the region 9q22-33 [3, 5, 8]; however the SNP and haplotype frequencies found within each population indicate multiple risk alleles for such a complex disease, due in part to maternal or paternal overtransmission [3]. Moreover, as its role with the expression of TG and TPO suggests, mutations at the* FOXE1 *locus in 9q22 have been associated with thyroid disorders such as congenital HT due to thyroid dysgenesis, primary HT, goiters, nonmedullary thyroid cancer, PTC, and TC [5, 8].

*FOXE1* is responsible for palatogenesis, and it appears that casual mutations in noncoding regions that regulate* FOXE1* expression are related to CL and CP [3], such as SNPs rs7850258, rs12342417, and rs10984103 within the* FOXE1* locus that have been associated with high risk for orofacial clefting [8].

Few studies have been carried out on Mexican population or in persons with American-Indian ethnicity; however, research has shown that SNP rs4460498 and rs375829, located in the* FOXE1* locus, are associated with nonsyndromic CP and CL for European and Mayan-Mesoamerican descendants [7]. This study obtained important significance in high risk of CL/CP for rs4460498 *p*_Europe_ = 6.50 × 10^−6^ and *p*_Mayan_ = 0.051, and for rs375829 *p*_Europe_ = 2.41 × 10^−5^ and *p*_Mayan_ = 0.0299.

### 3.4. Relation of* FOXE1* with Hypothyroidism

HT is the most common thyroid disorder characterized by deficiencies of thyroid hormones T3 and T4 (triiodothyronine and thyroxine, resp.), which are regulators of metabolism and development. A common marker of HT is the high levels of Thyroid-Stimulating Hormone (TSH), which indicate impaired thyroid function; however, in other cases, reduced levels of TSH cause low levels of T3 and T4. Iodine deficiency is the most common cause of HT, but congenital HT and autoimmune HT are other varieties with different genetic causes [20] that cause central HT, primary HT, alterations in thyroid transcription factors (with which the* FOXE1* gene is associated), dyshormonogenesis, and other genes such as the* TG* and* TSH* genes [19].

A mutation in the coding sequence of* FOXE1* that shifts Arginine-102 to a Cysteine (R102C), a highly conserved residue within the forkhead domain of the protein, impairs* FOXE1* from binding DNA; thus, it is inactive toward transcription. This inactivity leads to thyroid dysgenesis in the patient, which causes congenital HT, CP, spikey hair, and bilateral choanal atresia. Interestingly, because athyreosis is not present, human thyroid formation and development do not depend entirely on* FOXE1* activity [21].

SNPs that are present in other autoimmune diseases, such as rs6679677 near* PTPN22*, rs3184504 in* SH2B3*, and rs2517532 in HLA class I region, are linked to HT, while rs4915077 near* VAV3* [20] and other SNPs such as rs7850258, rs965513, rs925489, and rs10759944, all located near* FOXE1* in chromosome 9, exhibit genome-wide significance with primary HT [18]. Other SNPs in chromosome 9 that appear to be associated with primary HT are rs4979402, rs4979397, rs1408528, and rs1535971, located in* DFNB31* that is 16.6 Mb from* FOXE1* and not in LD with other SNP of* FOXE1*; however, these later regions do not correlate when TSH is not expressed [18].

### 3.5. Relation of* FOXE1* with Thyroid Cancer

TC is a complex polygenic disorder and the networking of the molecular mechanisms involved in its development, the genes involved in the process, and the risk environmental factors remain to be understood. It has been proposed that TC is the result of multiple low-to moderate penetrance genes interacting with each other, acting together with other transcriptional regulators that produce loss- or gain-of-function and that, together with environmental and other genetic risk factors, can lead to the development of cancer [6, 14, 21]. However, this explains only a small portion of the incidence, as there are other genetic factors involved. TC types, such as FTC, Differentiated Thyroid Cancer (DTC, which includes PTC and MTC), and undifferentiated anaplastic thyroid carcinomas, have different biomarkers and cellular conformations and thus different molecular pathways involved in their development.

rs7850258, located in the* FOXE1* promoter region near craniofacial enhancer hsCNE-67, has direct involvement in the development of thyroid and heart, and is also associated with HT and TC, as it was found to alter the enhancer function in both oral epithelial and thyroid cell lines. In this SNP, allele G is associated with HT and CL, whereas allele A is associated with TC [5].

Studies in several countries [6, 9–11, 14–16] have revealed that* FOXE1* locus polymorphisms in chromosome 9q22.33, such as rs965513 and polymorphisms (rs7849497, rs1867277, rs1867278, rs1867279, and rs1867280) in the promoter regions for this factor are associated with an increased risk of nonmedullary thyroid cancer, differentiated thyroid cancer, and the Bamforth–Lazarus syndrome, in which the mutation of a serine-57 to asparagine (S57N) in the forkhead-DNA binding domain of* FOXE1* leads to CP, choanal atresia, bifid epiglottis, thyroid agenesis or dysgenesis, hypothyroidism, and spikey hair [22]. Although there is no clear evidence of the role of the rs1867279 and rs1867280 in CL, HT, and PTC pathogenesis, functional prediction analysis showed that both SNPs could modify the transcription factor binding site and thus modulate the expression of relevant genes in the thyroid morphogenesis. Otherwise, the rs1867279 and rs1867280 might be in LD with the true casual variant, such as rs1867277, because a high LD has been described in the* FOXE1* region. On this regard, it has been reported that the rs1867277A allele recruits the USF1/USF2 transcription factors and has been shown to be involved in an allele-specific transcriptional regulation of* FOXE1* in thyroid cancer [6]. Actually, the rs1867277 have been found related to TC in several populations [21, 23, 24].

The information about the association of the* FOXE1* SNP rs1867277 with TC appears to vary with ethnicity and cancer type [16, 23]. For example Bychkov et al. [12] found overexpression of rs1867277 and rs965513 by immunohistochemical staining in PTC in Japanese persons. On the contrary, neither Maillard et al. [14] in French Polynesians nor Bonora et al. [25] in European descendants found an association of this SNP with nonmedullary thyroid cancer.

SNP rs1867277, within the −283G>A promoter region, was assessed functionally by Landa et al. [6] and their results suggest that this SNP increases the transcriptional activity of the* FOXE1* gene promoter by recruitment of leucine zipper USF 1 and 2. Thus, it is likely that recruiting USF1 and USF2 proteins produces a deregulation of* FOXE1 *that leads to malignant behavior of thyroid cells [9].

It is been demonstrated that, through a number of signalling pathways,* FOXE1*, along with the remainder of the family of FOX transcription factors, can induce* WNT5A*, a gene (located in 3p14.3) expressed in a variety of human tumors [26]. Thus, by deregulation of* FOXE1*, it is likely that this protein can become a tumor suppressor or an oncogene through the Wnt pathway, either dependently or independently of *β*-catenin [17]. Also, there is evidence that the* RET/PTC3* protooncogene that causes TC decreases* FOXE1* expression. Similarly, in squamous cell carcinoma, the 9q22 region of the chromosome (where* FOXE1 *is localized) is lost and, in other cancers, hypermethylation of promoter is also observed [5].

Levels of* FOXE1 *and* PAX8* messenger RNA (mRNA) in thyroid nodules have been found to be decreased in malignant thyroid lesions and higher in benign lesions, and even the gene expression patterns of* FOXE1* and* PAX8* can help in differentiating FTC from MTC and the Follicular Variant of Papillary Thyroid Carcinoma (FVPTC) [27].

Because we did not sequence the entire gene, our analysis did not exclude the hypothesis that* FOXE1* mutations contribute to the development of these diseases. Numerous evidences have been published suggesting that this gene is involved in these three diseases. In the same haplotype analysis of the members of a Portuguese family with nonmedullary thyroid carcinoma, the selected individuals with the disease not only shared the* FOXE1 p.A248G* variant, but also the same haplotype between markers rs965512 and D99S180 (located in chromosome 9), including rs1867279 and rs1867280 (SNPs present in our patient). However, these persons did not exhibit an association with any of the other polymorphic loci with the cancer because, as according to the authors and as reported in a previous paper, 15% of Portuguese control population have these SNP [17]. However, we propose that ethnicity and environmental factors are very important in triggering a disruptive response in rs1867279 and rs1867280, as in the case of our patient, in whom these SNPs are acting together, along with other risk factors (ethnic and environmental), to produce CL, HT, and TC.

Given the puzzling findings reported with rs1867277 [12, 16, 22] in* FOXE1* or rs944289 in* NKX2* [15], it is obvious that ethnicity and environmental factors play a key role for the development of TC and other malignancies. Additionally, the involvement of* FOXE1 *in thyroid formation and function, as well as the formation of craniofacial structures, relates mutations in the noncoding region of* FOXE1* to the phenotype of our patient.

Evidence such as the Bamforth–Lazarus syndrome [22], or mutations in* FOXE1* that explain an association of hypothyroidism and cleft palate [28], in addition to observational and comparative data of human and animal models null for* Foxe1* [5, 29] that are consistent with CP, CL, and other thyroid abnormalities, suggests that mutations or SNP in* FOXE1* can be a common cause for a possible new syndrome characterized by CL, HT, and PTC. In this line, Tables 2 and 3 illustrate some of the most studied SNPs and genes probably associated with these diseases.

Seeking an explanation for the possible identification of the syndrome that has been discussed, the* FOXE1* mutation has been tested, being negative as far as could be analysed. A possible candidate gene in this situation is E-cadherin* CDH1* on 16q22.1, which is also expressed in critical stages of lip and palate development in embryos [30, 31]. Hence, there are many more genetic alterations that could explain this possible association.

## 4. Conclusions

The association of CL, HT, and PTC might be component of a new syndrome; however* FOXE1* coding region, which has been involved with these entities, has not exhibited mutations or SNPs. Based on this case study and a review of the literature on the role that the* FOXE1 *gene plays in these pathologies, a more extensive sequence analysis (including other genes) is needed to rule out its role in the above-mentioned diseases.

## Figures and Tables

**Table 1:** Sequence of the primers used.

ID	Sequence	Length	Tm	Amplicon
1F	GTCACTCCCGAGCCTCTGT	19	60.41	395 pb
1R	GTAGCTGTAGGGCGGCTTC	19	59.99	

2F	AGCCGCCCTACAGCTACAT	19	59.87	488 pb
2R	CGCGGGGTAGTAGACTGG	18	59.26	

3F	GCCGTCTATGCAGGCTACG	19	61.8	639 pb
3R	GGGTCCCAGTTGAGTCCTCT	20	60.5	

F: forward; R: reverse.

**Table 2:** SNPs found in *FOXE1* related to orofacial clefts: cleft lip (CL) and cleft palate (CP), papillary thyroid cancer, and hypothyroidism.

Pathology	SNP in* FOXE1* or in its promoter regions	Sample/population
Orofacial clefts CL/P	rs4460498	Americans, Colombians, Danish, Filipinos, Norwegian [3].
	rs894673	Americans, Colombians, Filipinos [3].
	rs3758249	African-Brazilians, Americans, Central-Europeans, Colombians, Danish, Filipinos, Mayan-Mesoamerican, Norwegian [3, 4, 7].
	rs1867278	Americans, Filipinos, Norwegians, Danish [3, 4].
	rs1867280	Americans, Colombians, Danish, Filipinos, Norwegian [3].
	rs7850258	Human fetal oral epithelial thyroid cell line, Caucasian Europeans, Hondurans [3, 5].
	rs12342417	Europeans [5, 7].
	rs10984103	Europeans, Filipinos [8].
	rs4460498	Central-Europeans, Mayan-Mesoamerican [8].
Papillary thyroid cancer	rs1867277	Caucasian Australians, Italians, Japanese, Portuguese, Spanish, Turkish [6, 9–12].
	rs7850258	Rat FRTL epithelial thyroid cell line, zebra fish, mouse [5].
	rs965513	Colombians, European descents, Germans, Icelandics, Japanese, Polynesians, Portuguese [12–16].
	rs894673	Turkish [10].
	rs3758249	Caucasian Australians, Turkish [10, 11].
	rs907577	Caucasian Australians [11].
	rs3021526	Caucasian Australians [11].
	rs1443434	Caucasian Australians [11].
	rs907580	Caucasian Australians [11].
	Rs7849497	Portuguese [13].
	Rs1867278	Portuguese [13].
	Rs1867279	Portuguese [13].
	Rs1867280	Portuguese [13].
Hypothyroidism	rs7850258	Rat FRTL epithelial thyroid cell line, zebra fish, mouse, European [5, 17].
	rs965513	Europeans [17].
	rs925489	Europeans [17].
	rs10759944	Europeans [17].

**Table 3:** Genes related to orofacial clefts: cleft lip (CL) and cleft palate (CP), papillary thyroid cancer, or hypothyroidism.

Pathology	Genes
Orofacial clefts CL/P	*BMP4* *CRISPLD2* *FGF8* *FGFR1* *FGFR2* *FOXE1* *GLI2* *GSTM1* *GSTT1* *JAG2* *LHX8* *MSX1* *MSX1* *MSX2* *NAT1* *NAT2* *PTCH* *PVRL1* *RYK * *SATB2* *SKI* *SPRY2* *TBX10* *TGFB3*
Papillary thyroid cancer	*ATM* *NKX2* *RET *(RET/PTC rearrangement)
Hypothyroidism	*DFNB31* *PTPN22* *SH2B3* *VAV3*
